# Proteomic Profiling of Astrocytic O-GlcNAc Transferase-Related Proteins in the Medial Prefrontal Cortex

**DOI:** 10.3389/fnmol.2021.729975

**Published:** 2021-11-02

**Authors:** Jun Fan, Qiu-Ling Zhong, Ran Mo, Cheng-Lin Lu, Jing Ren, Jia-Wen Mo, Fang Guo, You-Lu Wen, Xiong Cao

**Affiliations:** ^1^Department of Neurobiology, School of Basic Medical Sciences, Southern Medical University, Guangzhou, China; ^2^Key Laboratory of Mental Health of the Ministry of Education, Southern Medical University, Guangzhou, China; ^3^Guangdong-Hong Kong-Macao Greater Bay Area Center for Brain Science and Brain-Inspired Intelligence, Southern Medical University, Guangzhou, China; ^4^Guangdong Province Key Laboratory of Psychiatric Disorders, Southern Medical University, Guangzhou, China; ^5^Department of Anesthesia, Guangzhou Women and Children’s Medical Center, Guangzhou Medical University, Guangzhou, China; ^6^Department of Psychology and Behavior, Guangdong 999 Brain Hospital, Guangzhou, China; ^7^National Demonstration Center for Experimental Education of Basic Medical Sciences, Southern Medical University, Guangzhou, China

**Keywords:** O-GlcNAc transferase, astrocyte, medial prefrontal cortex, proteomic, post-translational modification

## Abstract

The medial prefrontal cortex (mPFC), a key part of the brain networks that are closely related to the regulation of behavior, acts as a key regulator in emotion, social cognition, and decision making. Astrocytes are the majority cell type of glial cells, which play a significant role in a number of processes and establish a suitable environment for the functioning of neurons, including the brain energy metabolism. Astrocyte’s dysfunction in the mPFC has been implicated in various neuropsychiatric disorders. Glucose is a major energy source in the brain. In glucose metabolism, part of glucose is used to convert UDP-GlcNAc as a donor molecule for O-GlcNAcylation, which is controlled by a group of enzymes, O-GlcNAc transferase enzyme (OGT), and O-GlcNAcase (OGA). However, the role of O-GlcNAcylation in astrocytes is almost completely unknown. Our research showed that astrocytic OGT could influence the expression of proteins in the mPFC. Most of these altered proteins participate in metabolic processes, transferase activity, and biosynthetic processes. GFAP, an astrocyte maker, was increased after OGT deletion. These results provide a framework for further study on the role of astrocytic OGT/O-GlcNAcylation in the mPFC.

## Introduction

The medial prefrontal cortex (mPFC) comprises a crucial element of the brain networks that have been implicated in the regulation of behavior and autonomic responses to stress (Heidbreder and Groenewegen, 2003). Astrocytes, the most abundant glial cell subtype, serve to establish a suitable environment for the functioning of neurons. Astrocytes have been identified as exerting a wide variety of physiological functions that range from structural and metabolic support to the modulation of synaptic transmission and information processing (Nedergaard et al., 2003; Volterra and Meldolesi, 2005). Astrocyte dysregulation in the mPFC has been implicated in various neuropsychiatric disorders, including major depressive disorder (MDD), anxiety disorder, epilepsy, and learning and memory deficits, among others (Ongur et al., 1998; Bicks et al., 2015; Li et al., 2021).

Glucose is the major energy source for the brain. A deficit in glucose metabolism leads to an increased risk of neuropsychiatric diseases, such as depression and anxiety (Mergenthaler et al., 2013). Others and we have previously shown that astrocyte-derived ATP and lactate can modulate mood disorders and fear memory (Cao et al., 2013; Kambe et al., 2021; Yin et al., 2021). Although most glucose is used to generate ATP and lactate, during glucose metabolism, some of the available glucose is used for the biosynthesis of UDP-N-acetylglucosamine (UDP-GlcNAc), which serves as a donor molecule for O-GlcNAcylation. O-GlcNAcylation is a dynamic post-translational modification (PTM) that fine-tunes multiple processes governing fundamental cellular processes, such as signal transduction, transcription, translation, and proteasomal degradation (Hart et al., 2007; Yang and Qian, 2017; Lagerlof, 2018). The enzyme O-GlcNAc transferase (OGT) catalyzes the covalent attachment of N-acetyl-D-glucosamine to serine or threonine residues of proteins, whereas β-N-acetylhexosaminidase (also known as O-GlcNAcase [OGA]) catalyzes its removal. OGT is ubiquitously expressed and participates in specialized neuronal processes in different brain regions (Ruan et al., 2014; Zhang et al., 2014; Lagerlof et al., 2016), including synapse maturation, sensory neuron survival, and the regulation of feeding behavior in the paraventricular nucleus (Taylor et al., 2014; Lagerlof et al., 2016, 2017; Su and Schwarz, 2017). OGT also acts as a stress sensor. For example, OGT is an important placental biomarker of maternal stress, while reduced placental OGT levels exert long-term effects on metabolism and neurodevelopmental programming through disrupting the hypothalamic–pituitary–adrenal (HPA) axis (Howerton et al., 2013; Howerton and Bale, 2014). Astrocytes are perfectly positioned to balance the glucose metabolism in the brain, which are recognized as active players in brain energy delivery, production, storage, and providing substrate for many biological processes. However, relatively little is known about the role of OGT/O-GlcNAcylation in astrocytes in the brain.

Here, we generated astrocyte specific conditional *ogt* knockout mice, tissues of mPFC were collected, and we undertook a liquid chromatography–tandem mass spectrometry (LC–MS/MS)-based proteomics. Through bioinformatics analysis, we identified several astrocytic OGT associated metabolic processes, channel activities, and biosynthetic processes, as well as several KEGG pathways. Moreover, GFAP, a marker of reactive astrocytes, was modulated by OGT in the mPFC. The results are expected to increase our understanding of the molecular mechanisms underlying the role of OGT in astrocytes of the mPFC.

## Materials and Methods

### Animals

Four to five mice were socially housed and maintained under standard housing conditions on corn cob litter in a temperature (23 ± 1°C) and humidity (40%) controlled animal room on a 12 h light/dark cycle (lights were on from 07:00 to 19:00 every day) with free access to food and water.

*OGT*^*flox/flox*^ mice were purchased from Jackson Laboratories (Stock No. 004860). *Fgfr3-iCreER^*T*2^* mice in a C57BL/6J background were generously provided by William D. Richardson (University College London). All experiments were reviewed and approved by the Animal Advisory Committee of Southern Medical University.

### Immunofluorescence Staining

Mice were transcardially perfused with saline followed by 4% paraformaldehyde (PFA) in 0.1 M phosphate-buffered saline (PBS), pH 7.4. The brains were removed, postfixed overnight in 4% PFA at 4°C, transferred to 30% sucrose in 0.1 M PBS, pH 7.4, and coronally sectioned (40 μm) on a cryostat (Leica CM3000). After washing three times with PBS, the sections were incubated in blocking buffer containing 5% normal goat serum in 0.2% Triton X-100/PBS (PBST) for 1 h at room temperature. Then, with primary antibodies against OGT (Abcam, ab96718, 1:250, RRID: AB_10680015), glial fibrillary acidic protein (GFAP) (Cell Signaling Technology, 3670, 1:300, RRID: AB_561049), and NeuN (Millipore, MAB377, 1:500, RRID: AB_2298772) in blocking buffer overnight at 4°C. After washing, the samples were incubated with secondary antibody (Invitrogen, A11032, 11034, 11037, 11077, RRID: AB_2576217, AB_2534095, AB_2534091, and AB_2534121) for 1 h at room temperature. Nuclei were counterstained with DAPI. Samples were mounted on glass slides and then cover slipped with anti-fade solution, visualized using a Nikon fluorescence microscope. Mean intensity levels of GFAP were measured by ImageJ.

### Cell Culture

Tissues isolated from the cerebral cortex of mice on postnatal day 1 were washed in ice-cold PBS, transferred to a 50-mL tube containing 0.5 mL of PBS, and dissociated using a pair of sterile operating scissors. The samples were then incubated with 0.25% trypsin (Invitrogen) in 0.5 mM EDTA at 37°C for 10 min. Culture medium was added to neutralize the trypsin and the cell suspension was transferred into 15-mL tubes and centrifuged at 6,000× *g* for 6 min. The pellet was resuspended in 10 mL of culture medium (Dulbecco’s modified Eagle’s medium [DMEM] F12 + 10% fetal bovine serum [FBS]). The cells were placed in a culture flask at a density of 5 × 10^6^ cells per 5 mL and incubated in a humidified incubator with 5% O_2_ and 95% CO_2_ at 37°C.

### Western Blotting

Cultured cells and mouse brain tissues were homogenized in lysis buffer (RIPA) with the presence of protease (PMSF) and phosphatase inhibitors (Sigma) on ice for 30 min and centrifuged at 13,000 rpm for 20 min at 4°C. Protein samples (30–60 μg) were resolved by 10% sodium dodecyl sulfate-polyacrylamide gel electrophoresis (SDS–PAGE) and subsequently immunoblotted onto polyvinylidene difluoride (PVDF) membranes (Millipore) in ice-cold buffer (25 mM Tris HCl, 192 mM glycine, and 20% methanol) by electrotransfer for 2 h. The membranes were incubated with the indicated antibodies (OGT: Abcam, 1:1,000, RRID: AB_10680015; GFAP: CST 1:1,000, RRID: AB_561049; and GAPDH: Proteintech, 1:5,000, RRID: AB_2107436) at 4°C overnight. Then, with HRP-conjugated goat anti-rabbit mouse or HRP-conjugated goat anti-rabbit (Proteintech, SA00001-1, SA00001-2, 1:5,000, RRID: AB_2722564 and AB_2722565) secondary antibodies were then applied and incubated at room temperature for 1 h. Immunoreactive bands were visualized using an enhanced chemiluminescence reagent (Thermo Fisher) on a ChemiDoc XRS Gel Imaging System (Bio-Rad). Band intensities of the Western blotting images were analyzed using Image Lab (Bio-Rad). The blots were cropped according to their molecular weight. Tissue levels of GAPDH were used as loading controls. Protein expression was normalized to the control levels.

### Protein Extraction for Proteomics

The mPFC sample was ground into powder in liquid nitrogen and then transferred to a 5-mL centrifuge tube. Four volumes of lysis buffer (8 M urea, 1% protease inhibitor cocktail) were then added to the powder, followed by three rounds of sonication on ice using a high-intensity ultrasonic processor (Scientz). The remaining debris was removed by centrifugation at 12,000× *g* at 4°C for 10 min. Finally, the supernatant was collected, and the protein concentration was determined using a bicinchoninic acid assay (BCA) kit (Thermo Scientific) according to the manufacturer’s instructions.

### Trypsin Digestion

The protein solution was reduced with 5 mM dithiothreitol for 30 min at 56°C and alkylated with 11 mM iodoacetamide for 15 min at room temperature in the dark. The protein sample was then diluted to a urea concentration of less than 2 M by adding 100 mM TEAB. Finally, trypsin was added at a 1:50 trypsin-to-protein mass ratio for the first digestion (overnight) and a 1:100 trypsin-to-protein mass ratio for a second 4-h digestion.

### TMT/iTRAQ Labeling

After trypsin digestion, the peptides were desalted by Strata X C18 SPE column (Phenomenex) and vacuum dried. The peptides were reconstituted in 0.5 M TEAB and processed according to the protocol of the TMT kit/iTRAQ kit manufacturer. Briefly, one unit of TMT/iTRAQ reagent was thawed and reconstituted in acetonitrile. The peptide mixtures were then incubated for 2 h at room temperature, pooled, desalted, and dried by vacuum centrifugation.

### High-Performance Liquid Chromatography Fractionation

The tryptic peptides were fractionated by high-pH, reverse-phase high-performance liquid chromatography (HPLC) using a Thermo Betasil C18 column (5 μm particle size, 10 mm I.D., 250 mm length). Briefly, peptides were first separated into 60 fractions via an 8–32% acetonitrile gradient (pH 9.0) over 60 min and then combined into 6 fractions and dried by vacuum centrifugation.

### LC–MS/MS Analysis

The tryptic peptides were dissolved in 0.1% formic acid (solvent A), directly loaded onto a homemade, reversed-phase analytical column (15 cm length, 75 μm i.d.). The gradient consisted of an increase from 6 to 23% solvent B (0.1% formic acid in 98% acetonitrile) over 26 min, 23–35% over 8 min, climbing to 80% in 3 min, and then holding at 80% for the last 3 min. All at a constant flow rate of 400 nL min^–1^ on an EASY-nLC 1,000 ultraperformance liquid chromatography (UPLC) system. The peptides were subjected to nanospray ionization (NSI) followed by MS/MS in a Q Exactive Plus (Thermo) coupled online to the UPLC. The electrospray voltage applied was 2.0 kV. The *m*/*z* scan range was 350–1,800 for a full scan, and intact peptides were detected in the Orbitrap at a resolution of 70,000. The peptides were then selected for MS/MS with the normalized collision energy (NCE) set at 28 and the fragments were detected in the Orbitrap at a resolution of 17,500. The data-dependent procedure alternated between one MS scan followed by 20 MS/MS scans with 15.0 s dynamic exclusion. Automatic gain control (AGC) was set at 5^E4^. Fixed first mass was set as 100 *m*/*z*.

### Database Search

The resulting MS/MS data were processed using MaxQuant software with an integrated Andromeda search engine (v.1.5.2.8). Tandem mass spectra were searched against the human UniProt database concatenated with a reverse decoy database. Trypsin/P was specified as the cleavage enzyme with up to four missing cleavages allowed. The mass tolerance for precursor ions was set as 20 ppm in First search and 5 ppm in Main search, and the mass tolerance for fragment ions was set as 0.02 Da. Carbamidomethyl on Cys was specified as the fixed modification, while acetylation modification and oxidation on Met were specified as the variable modifications. The false discovery rate (FDR) was adjusted to < 1% and the minimum score for modified peptides was set at > 40.

### Gene Ontology Annotation and Enrichment Analysis

The Gene Ontology (GO) annotation proteome was derived from the UniProt-GOA database^1^. Identified protein IDs were first converted to UniProt IDs and then mapped to GO IDs by protein ID. When an identified protein was not annotated by the UniProt-GOA database, InterProScan was used to annotate protein function based on sequence homology with GO-annotated proteins. Finally, the proteins were classified by GO annotation into three categories: biological process, cellular component, and molecular function. For each category, a two-tailed Fisher’s exact test was employed to test the enrichment of each differentially expressed protein against all identified proteins. A corrected *p*-value < 0.05 was considered significant.

### Domain Annotation

Domain functional descriptions for the identified proteins were annotated by InterProScan (InterPro database)^2^ based on protein sequence homology.

### Kyoto Encyclopedia of Genes and Genomes Pathway Annotation and Enrichment Analysis

For protein pathway annotation, the KEGG online service tools KAAS and KEGG mapper were used to annotate each protein’s KEGG database description and map the annotation results on the KEGG pathway database, respectively. For pathway enrichment analysis, a two-tailed Fisher’s exact test was used to test the enrichment of each differentially expressed protein against all identified proteins. Pathways with a corrected *p*-value < 0.05 were considered significant and were classified into hierarchical categories according to the KEGG website.

### Subcellular Localization

WoLF PSORT^3^, an updated version of PSORT II used for the prediction of eukaryotic sequences, was utilized to predict subcellular localization. For protokaryon species, subcellular localization prediction was performed using CELLO^4^.

### Co-expression Network

All the accession numbers or sequences of the differentially expressed proteins were searched against the STRING database (version 10.1)^5^ for protein–protein interactions (PPI). Only interactions between the proteins belonging to the searched data set were selected, thereby excluding external candidates. STRING defines a metric called “confidence score” to define interaction confidence, and we fetched all interactions with a confidence score of ≥ 0.7 (high confidence). The generated PPI network was visualized in the R package “networkD3.”

### Statistical Analysis

All experiments and data analyses were conducted in a blinded manner. The number of replicates (*n*) is indicated in the figure legends. Data are presented as means ± SEM. Statistical analysis was performed using SPSS 20.0 with the appropriate inferential methods. Normally distributed data were tested by two-sided, unpaired *t*-tests for two-group comparisons. Statistical significance was set at *p* < 0.05.

## Results

### Generation of Astrocyte-Specific *Ogt* Conditional KO Mice

**FIGURE 1:**
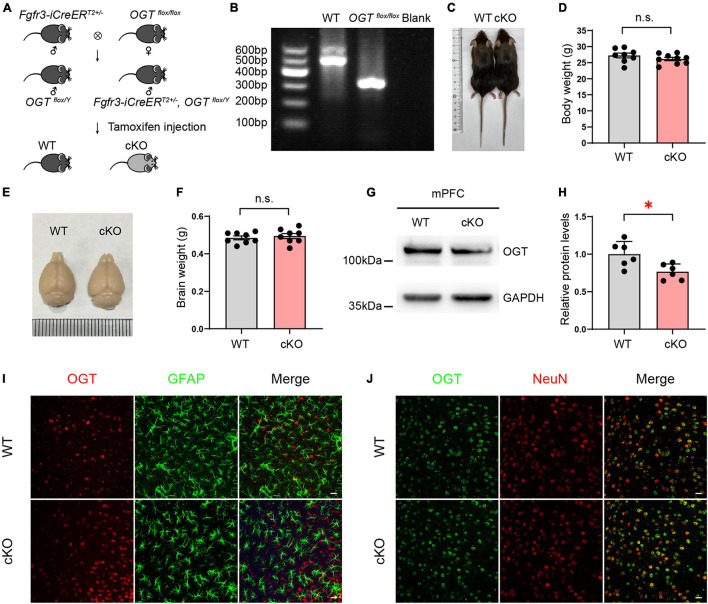
Generation of astrocyte-specific OGT conditional knockout mice. **(A)** Generation of OGT cKO mice by crossing *OGT*^*flox/flox*^ lines with *Fgfr3-iCreER^*T*2^* lines. **(B)** Genotyping of *OGT*^*flox/flox*^ mice and littermate wide-type mice. **(C,D)** The represented image **(C)** and quantification of body weight changes after TAM injection **(D)** for cKO and littermate controls. *n* = 8 (WT), 9 (cKO). **(E,F)** Representative image **(E)** and the quantification of brain weight change **(F)** after TAM injection. *n* = 8 (WT), 8 (cKO). **(G,H)** Western blot images **(G)** and quantification **(H)** of OGT levels in the mPFC of cKO and WT mice. *n* = 6 (WT), 6 (cKO). **(I)** Double immunofluorescence staining of OGT (red) with GFAP (green) in cKO and WT mice. Scale bar = 20 μm. **(J)** Double immunofluorescence staining of OGT (green) with NeuN (red) in cKO and WT mice. Scale bar = 20 μm. All data are presented the mean ± SEM. Two-sided unpaired *t*-test, ^∗^*P* < 0.05; n.s, no significance.

*Fgfr3-iCreER^*T*2^*, *OGT*^*flox/Y*^ mice were generated by crossing *OGT*^*flox/flox*^ mice with *Fgfr3*-*iCreER^*T*2^* mice. These mice can specifically express recombinant Cre recombination in adult astrocytes following tamoxifen (TAM) injection (Figures 1A,B). To excise the *loxp* sites by *Cre* recombination, 2-month-old male mice were intraperitoneally injected with TAM (Sigma) (100 mg kg^–1^ body weight) once a day for 5 consecutive days. TAM was dissolved in corn oil (Sigma) at a final concentration of 10 mg mL^–1^. Littermate *OGT*^*flox/Y*^ mice injected with TAM were used as controls. Four weeks after TAM injection, astrocyte-specific cKO animals exhibited growth rates and body size comparable with those of wild-type (WT) mice (Figures 1C,D), and the appearance and weight of the whole brain differed little between the two groups (Figures 1E,F). To examine the reduction of OGT in protein levels, tissues of mPFC in both cKO and WT mice were collected, Western blots were performed. The data showed that OGT levels were significantly reduced in the mPFC of cKO mice compared with littermate controls (Figures 1G,H). To confirm the specificity reduction in astrocytes, brain slices of mPFC were used for immunofluorescence staining in both WT and cKO mice. We employed a specific OGT antibody and co-stained with NeuN (a marker of neurons) or GFAP (a marker of astrocytes). The confocal images showed that the expressions of OGT in neurons were undifferentiated in the mPFC of WT and cKO mice. We co-stained mPFC tissue with antibodies targeting OGT and GFAP (an astrocyte marker) or NeuN (a neuron marker). Immunofluorescence revealed that GFAP-labeled astrocytes featured attenuated OGT staining in the mPFC of cKO mice compared with WT controls (Figure 1I), while the expressions of OGT in neurons were undifferentiated between the two groups (Figure 1J). These results proved that OGT cKO models were sufficient to reduce the OGT expressions in astrocytes but not in neurons.

### Protein Identification and Quantification in the Medial Prefrontal Cortex of Astrocyte-Specific *Ogt* KO Mice

**FIGURE 2:**
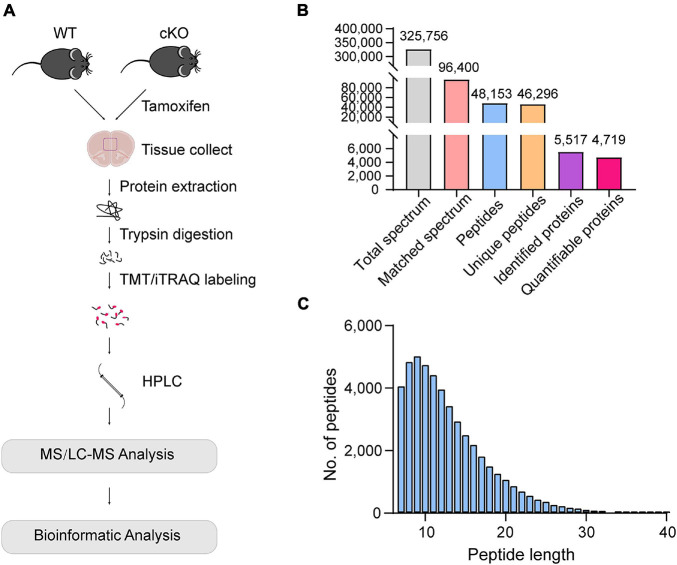
Protein identification and quantification in the mPFC of astrocyte-specific OGT knockout mice. **(A)** Schematic of the proteomics analysis in the mPFC of astrocytic OGT cKO and WT mice. **(B)** Basic information of MS data. **(C)** The distribution of peptide length.

To investigate the changes occurring in the proteome of OGT-depleted mPFC astrocytes, mPFC tissues of WT and *Ogt* cKO mice were collected and subjected to LC/MS after digestion and labeling (Figure 2A). A total of 325,756 spectra were identified and 96,400 were matched (Figure 2B). A total of 48,153 peptides had a length of approximately 7–13 amino acids (Figure 2C). Of these, 5517 were matched to known proteins and 4719 proteins were quantified (Figure 2B).

### Differentially Expressed Proteins in the Medial Prefrontal Cortex of Astrocyte-Specific *Ogt* KO Mice

**FIGURE 3:**
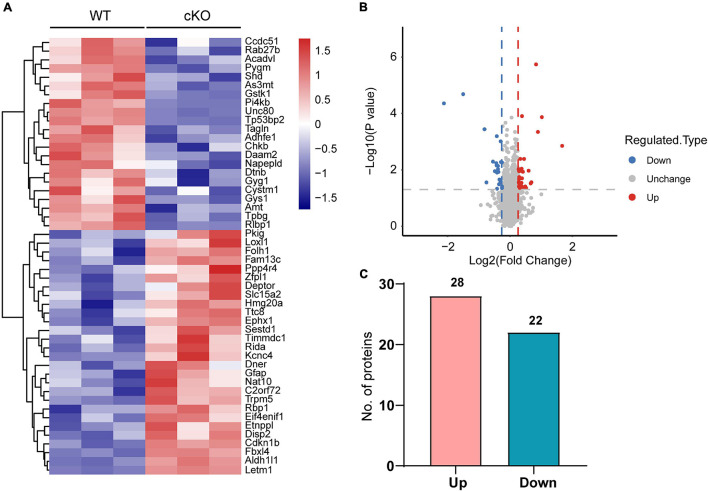
Differentially expressed proteins in the mPFC of astrocytic OGT knockout and WT mice. **(A,B)** Heatmap **(A)** and volcano plots **(B)** of differentially expressed proteins in the mPFC of astrocytic OGT knockout and WT mice. **(C)** Number of up- and downregulated proteins in the mPFC.

**FIGURE 4:**
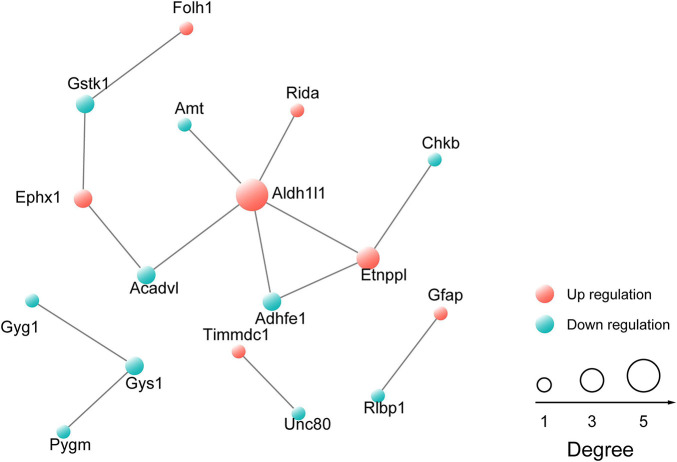
Protein-protein interaction (PPI) network of proteins that are differentially expressed in the mPFC. The network was generated using the STRING database. The red sphere represents upregulated proteins and the blue sphere represents downregulated proteins.

Next, differentially expressed proteins between the *Ogt* cKO and WT mice were screened out from the 4719 quantified proteins. A total of 50 proteins was found to be differentially expressed, including 28 that were upregulated and 22 downregulated in the mPFC of *Ogt* cKO mice (Figures 3A–C). The generated heatmap and volcano plots are shown in Figures 3A,B. Interestingly, GFAP and Aldh1l1, both of which are astrocyte markers, were among the upregulated proteins, indicating that OGT-related protein changes may be linked to astrocyte functions. Next, we generated a PPI network for the differentially expressed proteins and evaluated their possible regulatory effects (Figure 4). Aldh1l1 showed most regulatory effects with AMT, RIDA, and ETNPPl. GFAP also displayed significant interaction with RLBP1 (Figure 4).

### Gene Ontology Enrichment Analysis of Downregulated Proteins in the Medial Prefrontal Cortex

**FIGURE 5:**
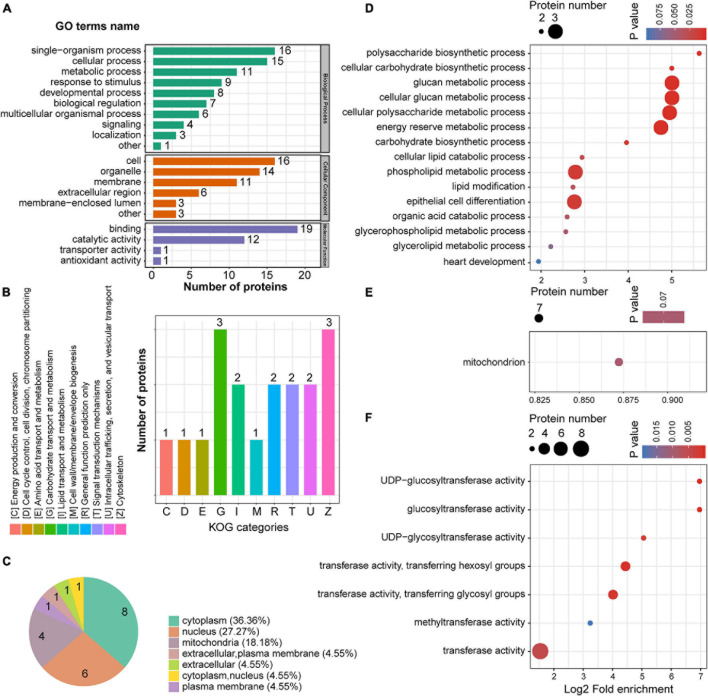
The GO enrichment of down-regulated proteins in the mPFC. **(A)** GO annotations of downregulated proteins in the mPFC. **(B)** Cluster of orthologous groups for enkaryotic complete genomes (KOG) classification of downregulated proteins. **(C)** Subcellular localization analysis of downregulated proteins. **(D–F)** GO enrichment analysis according to the downregulated proteins in the mPFC. Biological process **(D)**, cellular compartment **(E)**, and molecular function **(F)**.

The 22 downregulated proteins in the mPFC were successfully mapped with GO annotations and were classified into three ontologies containing 20 functional groups, including metabolic processes, response to stimulus, and binding, among others (Figure 5A). Clusters of orthologous groups for complete eukaryotic genomes (KOG) classification showed that the downregulated proteins were involved in multiple metabolic processes, including carbohydrate, lipid, and amino acid transport (Figure 5B). Most of the downregulated proteins were localized to the cytoplasm, nucleus, and mitochondria (Figure 5C). GO enrichment analysis was conducted for the 22 downregulated proteins in the mPFC. In the biological process category, metabolic processes such as glucan, cellular glucan, phospholipid, carbohydrate, and lipid metabolism were significantly enriched (Figure 5D), while in the cellular component, only mitochondrion was enriched among seven downregulated proteins (Figure 5E). In the molecular function, most of the enriched GO terms were related to transferase activity (Figure 5F).

### Gene Ontology Enrichment Analysis of Upregulated Proteins in the Medial Prefrontal Cortex

**FIGURE 6:**
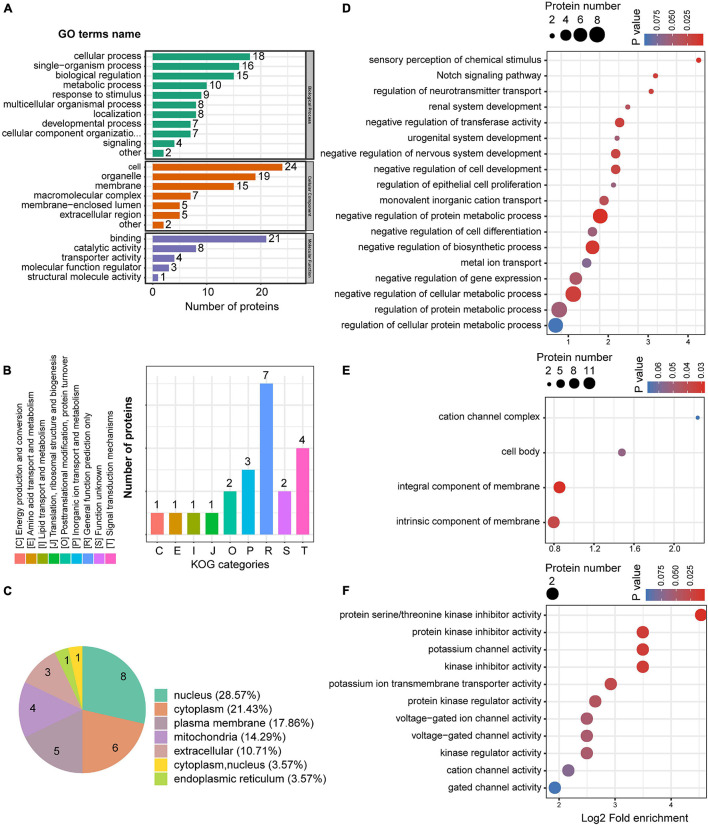
The GO enrichment of up-regulated proteins in the mPFC. **(A)** GO annotations of upregulated proteins in the mPFC. **(B)** Cluster of orthologous groups for enkaryotic complete genomes (KOG) classification of upregulated proteins. **(C)** Subcellular localization analysis of upregulated proteins. **(D–F)** GO enrichment analysis according to the upregulated proteins in the mPFC. Biological process **(D)**, cellular compartment **(E)**, and molecular function **(F)**.

We also conducted GO enrichment analysis for the 28 upregulated proteins. Twenty-three GO terms were classified into three ontologies, including cellular, biological, and metabolic processes (Figure 6A). KOG classification showed the greatest enrichment for inorganic ion transport and metabolism, while several upregulated proteins were also closely associated with signal transduction mechanisms (Figure 6B). Subcellular localization analysis showed that most of the upregulated proteins were localized to the nucleus, cytoplasm, plasma membrane, and mitochondria plasma membrane (Figure 6C). Notably, 17.86% of the upregulated proteins were located at the plasma membrane, whereas only 4.55% of the downregulated proteins showed a similar subcellular localization. GO enrichment analysis was also undertaken for the upregulated proteins. In the biological process, negative regulation of cell differentiation, transferase activity, biosynthetic process, and cellular metabolic processes were enriched among the upregulated proteins (Figure 6D). In the cellular component, intrinsic and integral components of a membrane were significantly enriched (Figure 6E), whereas channel activity, including potassium, voltage-gated ion, cation, and gate activities, were enriched in the molecular function (Figure 6F), as was protein serine/threonine kinase inhibitor activity.

### Construction of the Co-expression Network

**FIGURE 7:**
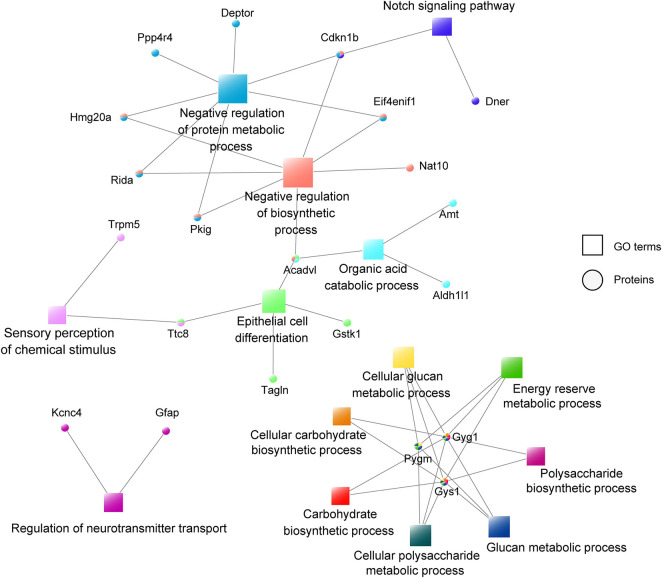
Co-expression network of the differentially expressed proteins and their enriched GO terms. The circular nodes represent the proteins and the square nodes represent the GO terms.

To further understand the function of astrocyte-specific OGT in the mPFC, a co-expression network was constructed according to the differentially expressed proteins and their enriched GO terms (Figure 7). A total of 28 upregulated and 22 downregulated proteins were uploaded to the protein network database and the interaction networks were determined with a minimum required interaction score of 0.700. The results showed that GO terms such as negative regulation of protein metabolic and biosynthetic processes, organic acid catabolic process, and epithelial cell differentiation were highly enriched. Moreover, three proteins (PYGM, GYG1, and GYS1) were associated with seven GO terms, including glucan, cellular glucan, cellular polysaccharide and energy reserve metabolic processes, and cellular carbohydrate and carbohydrate polysaccharide biosynthetic processes, indicating an important role for OGT in the regulation of metabolic and biosynthetic processes. Notably, GFAP was found to be involved in the regulation of the neurotransmitter transport, a process that is important for astrocyte/neuron communication (Figure 7).

### Kyoto Encyclopedia of Genes and Genomes Enrichment Analysis for the Differentially Expressed Proteins in the Medial Prefrontal Cortex

**FIGURE 8:**
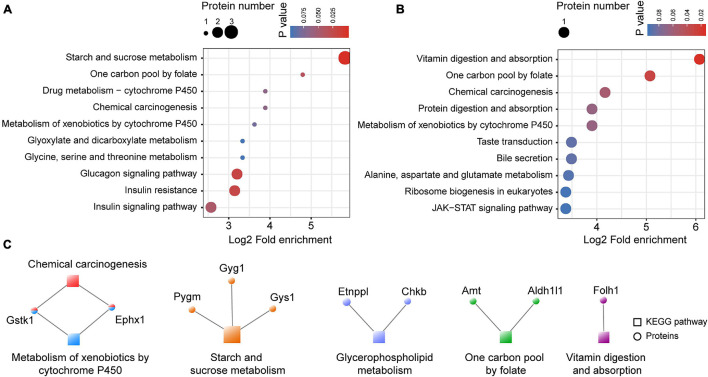
The KEGG enrichment of differentially expressed proteins in the mPFC. **(A)** The KEGG enrichment of downregulated proteins in the mPFC. **(B)** The KEGG enrichment of upregulated proteins in the mPFC. **(C)** Co-expression network of the differentially expressed proteins and their enriched KEGG pathways. The circular nodes represent the proteins and the square nodes represent the KEGG pathways.

KEGG enrichment analysis was performed for the proteins identified as being up- and downregulated in the astrocyte-specific *Ogt* cKO mice compared with the control mice. Metabolism of xenobiotics by cytochrome P450, one carbon pool by folate, and chemical carcinogenesis were enriched among both the up- and downregulated proteins. However, metabolism-related pathways, including starch and sucrose, glyoxylate and dicarboxylate, glycine, and serine and threonine metabolism, were only enriched among the downregulated proteins, as was insulin resistance and a glucose metabolism-related insulin signaling pathway (Figure 8A). Alanine, aspartate, and glutamate metabolism were only enriched among the upregulated proteins. Additionally, the JAK/STAT signaling pathway, which is activated by cytokines and growth factors and is well-conserved from *Drosophila* to mammals, was enriched among the upregulated proteins (Figure 8B). We also constructed a co-expression network of the differentially expressed proteins and their enriched GO terms. Three proteins (PYGM, GYG1, and GYS1) were enriched in the starch and sucrose metabolism pathway, which is highly associated with the regulation of many metabolic and biosynthetic processes (Figure 8C).

### Validation of the O-GlcNAc Transferase- Mediated Regulation of Glial Fibrillary Acidic Protein in Astrocytes

**FIGURE 9:**
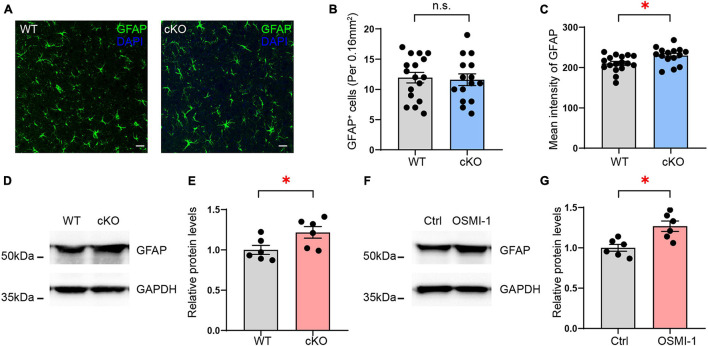
Validation of GFAP expression regulated by OGT in astrocytes. **(A)** Immunofluorescence staining of GFAP (green) in astrocyte-specific OGT conditional knockout mice and WT mice. Scale bar = 20 μm. **(B)** Quantification of GFAP positive cells in the mPFC of cKO and WT mice. *n* = 17 (WT), 15 (cKO). **(C)** Quantification of mean fluorescence intensity in the mPFC of cKO and WT mice. *n* = 17 (WT), 15 (cKO). **(D,E)** Western blot images **(D)** and quantification **(E)** of GFAP expression in the mPFC of astrocyte-specific OGT conditional knockout mice and controls. The blots were cropped according to their molecular weight. Tissue levels of GAPDH were used as loading controls. Protein expression was normalized to the control levels. *n* = 6 (WT), 6 (cKO). **(F,G)** Western blot images **(F)** and quantification **(G)** of GFAP levels in primary cultured astrocytes treated with OGT inhibitor (OSMI-1) and controls. *n* = 6 (Control), 6 (OSMI-1). Two-sided unpaired *t*-test. ^∗^*P* < 0.05; n.s: no significance.

GFAP, an astrocyte marker, was identified as being upregulated in the mPFC of astrocyte-specific *Ogt* cKO mice compared with control mice in the LC/MS analysis. To confirm the change in GFAP expression, we first performed GFAP staining in the mPFC of the *Ogt* cKO and WT mice and calculated the density of GFAP-positive cells. The results showed that the number of the GFAP-positive astrocytes was similar between the two groups (Figures 9A,B). However, we analyzed the intensity levels of GFAP in the mPFC. The data showed that the GFAP mean intensity levels were significantly higher in cKO mice than control groups (Figure 9C). These results indicated that OGT could modulate the expression of GFAP without affecting the number of astrocytes. Next, we employed Western blotting to measure GFAP expression in the mPFC and found that GFAP levels were significantly increased in the mPFC of astrocyte-specific *Ogt* cKO mice compared with that in the WT controls (Figures 9D,E). To further confirm that OGT did indeed regulate the expression of GFAP, we treated primary cultured astrocytes with 50 μM of the OGT inhibitor OSMI-1 for 4 h, collected total protein, and measured GFAP expression. The data showed that GFAP expression was increased after OSMI-1 treatment (Figures 9F,G), suggesting that OGT could modulate GFAP expression in astrocytes.

## Discussion

In this study, we identified a total of 4719 proteins in the mPFC by LC/MS, 50 of which were identified as being significantly altered after *Ogt* knockout in astrocytes, including 28 that were upregulated and 22 downregulated. Next, we performed GO and KEGG enrichment analysis for both the up- and downregulated proteins. The results showed that multiple metabolic processes and transferase activity were enriched among the downregulated proteins, whereas the upregulated proteins were enriched for a series of cell development processes and channel activities. The expression of GFAP, a well-documented marker for adult astrocytes, was increased in the mPFC of astrocyte-specific *Ogt* cKO mice compared with that in the controls; however, the total number of GFAP-positive astrocytes in the mPFC remained unaffected.

The mPFC is a key component of the brain network implicated in the regulation of social behavior, emotion, social cognition, and decision-making. The mPFC is divided into the medial precentral area, anterior cingulate cortex, prelimbic cortex, and infralimbic cortex subregions (Heidbreder and Groenewegen, 2003). More than 80% of neurons in the mPFC are pyramidal neurons (DeFelipe and Farinas, 1992; Kawaguchi, 2017). Neurons communicate with each other and serve as a central hub in the emotional circuits of the brain via the synapse, a tripartite structure consisting of the axon terminal of one neuron, the postsynaptic membrane of another, and the surrounding glial cell processes (Araque et al., 1999; Bicks et al., 2015). Astrocytes, the most abundant subtype of glial cell, are active players in this three-way communication. Astrocytes can remove excess K^+^ and glutamate following neuronal activity, processes that are essential for the maintenance of extracellular homeostasis and, consequently, proper functioning of the brain (Cheung et al., 2015). Moreover, astrocytes are increasingly thought to be involved in responses to stress, and have been found to play major roles in both the progression and repair of central nervous system (CNS) pathologies such as inflammation, epilepsy, ischemia, neurodegenerative diseases, and neurodevelopmental disorders (Allen and Barres, 2009; Sofroniew and Vinters, 2010).

O-GlcNAcylation is a dynamic PTM that regulates multiple processes governing fundamental cellular events, such as signal transduction, transcription, translation, and proteasomal degradation (Yang and Qian, 2017; Lagerlof, 2018). More than 4,000 proteins have been identified as containing O-GlcNAcylation sites, and are present widely in the cytoplasm, nucleus, plasma membrane, and mitochondria (Jayaprakash and Surolia, 2017). Disruption of O-GlcNAcylation is closely related to diabetes, cancer, and neurodegeneration (Ma and Hart, 2013; Dassanayaka and Jones, 2014; Singh et al., 2015; Peterson and Hart, 2016; Machacek et al., 2018). O-GlcNAcylation is mediated by the enzymes OGT and OGA, which, respectively, catalyze the covalent attachment of N-acetyl-D-glucosamine to serine or threonine residues of proteins and their removal. OGT was reported to be an important placental biomarker of maternal stress, while reduced placental OGT levels were shown to exert long-term effects on metabolism and neurodevelopmental programming through disrupting the HPA axis (Howerton and Bale, 2014). Astrocytes can rapidly respond to stress in the brain; however, whether OGT/O-GlcNAcylation affects these or any other responses in astrocytes remains largely unknown.

In our study, differentially expressed proteins in the mPFC of astrocyte-specific *Ogt* cKO mice were analyzed for molecular function and biological process ontologies using Blast2GO to understand the potential impact of these changes in protein expression. We found that the differentially expressed proteins were enriched for metabolic processes, transferase activity, and biosynthetic processes (Figures 4, 5). Notably, multiple metabolic processes, including those related to carbohydrate metabolism, lipid metabolism, and amino acid transport, were enriched among the downregulated proteins, while negative regulation of cellular protein metabolic process was enriched among the upregulated proteins. Astrocytes are perfectly positioned to balance metabolism in the brain. Their end-feet have rosette-like structures lying on the blood vessel wall, which allows direct exchange of metabolites between capillaries and nerve terminals (Kacem et al., 1998). Additionally, neurotransmitter receptors in astrocytes enable them to sense synaptic activity. Astrocytes communicate to neurons by releasing chemical messengers, including glutamate, ATP, lactates, neurotrophins, eicosanoids, and neuropeptides (Haydon, 2001; Newman, 2003; Henneberger et al., 2010). Disruption of the metabolic balance in the brain results in impaired brain functions. OGT acts as a metabolism sensor in the brain, and 24 h or longer of fasting can lead to a strong reduction in global O-GlcNAc levels in the hippocampus and cortex (Li et al., 2006). In our study, we found that OGT in astrocytes may contribute to the regulation of multiple metabolic processes. Moreover, channel activity was enriched among the upregulated proteins, including potassium, voltage-gated ion, cation, and gate channel activities. Potassium channels are widely distributed in the postnatal CNS. For example, Kir 4.1, which can regulate spatial K^+^ buffering and BDNF expression in astrocytes, contributes to the modulation of epilepsy, chronic pain, and depressive disorders (Ohno et al., 2018). Voltage-activated K^+^ (BK) channels are targeted to end-feet processes that wrap around brain parenchymal blood vessels where they play a critical role in the rapid dilation of intracerebral arterioles induced by adjacent neuronal activity (Contet et al., 2016). BK channels are downregulated in whole brain samples from mouse models of vascular amyloid deposition. Combined, these observations indicate that OGT might regulate channel activity in astrocytes.

GFAP is the hallmark intermediate filament protein in astrocytes. Its overexpression leads to a fatal encephalopathy characterized by the accumulation of the protein in Rosenthal fibers (Hol and Pekny, 2015). Reactive astrogliosis is a characteristic change in the morphology and function of astrocytes seen in many neurological disorders, including neurotrauma, ischemic stroke, and neurodegenerative diseases (Seifert et al., 2006; Burda and Sofroniew, 2014). GFAP is also considered to be a marker of reactive astrocytes. Here, we found that the expression of GFAP was significantly increased in the mPFC after *Ogt* KO in astrocytes, without a concomitant change in the numbers of GFAP-positive cells. Changes in GFAP expression were further confirmed through OGT inhibition in primary cultured astrocytes; however, whether the function of astrocytes was affected requires further exploration.

## Conclusion

In conclusion, our study showed that astrocytic OGT can influence the expression of proteins in the mPFC. Through bioinformatics analysis, we identified several metabolic processes, channel activities, and biosynthetic processes, as well as several KEGG pathways associated with *Ogt* KO in astrocytes. We further found that the expression of GFAP, an astrocyte marker, was increased following *Ogt* deletion. Although these results provide a framework for further study on the role of astrocytic OGT/O-GlcNAcylation in the mPFC, further studies are needed to validate the roles of these OGT-related proteins in brain function.

## Data Availability Statement

The datasets presented in this article are not readily available because the repository PRIDE where the data is hosted is not able to make the data public for technical reasons. Requests to access the datasets should be directed to the corresponding author.

## Ethics Statement

The animal study was reviewed and approved by Ethics Committee of Southern Medical University.

## Author Contributions

JF and Q-LZ performed most of the experiments, participated in the data analysis, and wrote the manuscript. JF, RM, Q-LZ, and C-LL performed the Western blotting and immunofluorescence staining. JR and Y-LW were responsible for cell culture. J-WM and RM carried out animal feeding and genotyping. FG performed the cell counting. XC supervised all phases of the project. All authors contributed to the article and approved the submitted version.

## Conflict of Interest

The authors declare that the research was conducted in the absence of any commercial or financial relationships that could be construed as a potential conflict of interest.

## Publisher’s Note

All claims expressed in this article are solely those of the authors and do not necessarily represent those of their affiliated organizations, or those of the publisher, the editors and the reviewers. Any product that may be evaluated in this article, or claim that may be made by its manufacturer, is not guaranteed or endorsed by the publisher.

## Footnotes

1
http://www.ebi.ac.uk/GOA/

2
http://www.ebi.ac.uk/interpro/

3
https://wolfpsort.hgc.jp/

4
http://cello.life.nctu.edu.tw/

5
https://string-db.org/
